# ASO Author Reflections: Applications of Artificial Intelligence in Oesophago-Gastric Malignancies—Present Work and Future Directions

**DOI:** 10.1245/s10434-021-10907-0

**Published:** 2021-11-18

**Authors:** Swathikan Chidambaram, Viknesh Sounderajah, Nick Maynard, Sheraz R. Markar

**Affiliations:** 1 Department of Surgery and Cancer, Imperial College London, London, UK; 2 Institute of Global Health Innovation, Imperial College London, London, UK; 3 Department of Surgery, Churchill Hospital, Oxford University Hospitals NHS Trust, Oxford, UK; 4 Department of Molecular Medicine and Surgery, Karolinska Institutet, Stockholm, Sweden

## Abstract

Our paper highlights the use of artificial intelligence (AI) in oesophageal and gastric malignancies with acceptable levels of accuracy for both diagnostic and surveillance purposes. Here, we comment on the past, present and future work necessary for incorporating AI into the clinical framework and practice.

## Past

Artificial intelligence (AI)-centred systems have shown promise in facilitating the diagnosis and management of many disease processes, including cancers.^1^ With an increase in the prevalence and mortality of oesophago-gastric (OG) cancers, there is a particular need for enhanced methods of diagnosis in this space, particularly as a means of surveillance. As such, there is much fervour around the potential of AI techniques to be able to recognise features of malignancy on routine investigations that this cohort of patients undergo, including endoscopy, computed tomography (CT) scans, CT-positron emission tomography (PET) scans and magnetic resonance imaging (MRI) scans.

## Present

Previous studies have shown varying degrees of success in developing AI systems to detect oesophageal and gastric cancers for both diagnostic and surveillance purposes. In our study, we have summarised their results and shown that there is considerable promise in this field, despite its relative nascency. In the quantitative synthesis, the pooled sensitivity and specificity for 1352 patients were 73.4% and 89.7%, respectively.^2^ Moreover, the incorporation of radiomic techniques can further enhance the diagnostic accuracy of the process. Key limitations in the field currently centre around the absence of a randomised clinical trial to evaluate performance prospectively, and the reliance upon small retrospective studies which are susceptible to bias.

## Future

There is clearly an abundance of potential for using AI in the management of OG cancers. Currently, we are in an era that is shifting towards an organ-sparing approach, relying solely on primary oncological treatment and avoiding the morbidity of surgical intervention. This is only possible due to effective chemo-radiotherapy (CRT) regimes developed in the past decade. The ongoing SANO and NEEDS trials are evaluating the safety and feasibility of this strategy.^3^ However, this is only possible if recurrence or residual cancer can be accurately detected on surveillance. A well-designed AI system that is trained and validated to achieve high diagnostic accuracies will play an unprecedented role in achieving this. To do this, large datasets of patient data, including clinical details, endoscopy images and imaging, need to be collated encompassing a range of factors including demographic (age, sex, ethnicity, comorbidities), oncological (CRT regime), surgical (approach, technique, extent of lymphadenectomy) and tumour level (histology, pre-operative and pathological TNM stages, grade). The AI system will also require robust independent validation using external datasets. Furthermore, the translation of the AI model to clinical practice requires infrastructure to be implemented, while also taking into consideration its cost-effectiveness as well as acceptability by stakeholders, including clinicians and patients. All of the above strongly emphasise the need for well-designed randomised clinical trials conducted on a large scale through collaboration to fully realise the potentials of artificial intelligence systems.
